# Macular outer nuclear layer, ellipsoid zone and outer photoreceptor segment band thickness, axial length and other determinants

**DOI:** 10.1038/s41598-023-32629-x

**Published:** 2023-04-03

**Authors:** Ya Xing Wang, Zhe Pan, Can Can Xue, Hui Xie, Xiaodong Wu, Jost B. Jonas

**Affiliations:** 1 Beijing Institute of Ophthalmology, Beijing Tongren Hospital, Beijing Ophthalmology and Visual Sciences Key Laboratory, Capital University of Medical Science, Beijing, China; 2 Department of Ophthalmology, Peking University Third Hospital, Beijing, China; 3 Department of Electrical and Computer Engineering, University of Iowa, Iowa City, USA; 4 Department of Ophthalmology, Medical Faculty Mannheim, Heidelberg University, Mannheim, Germany; 5 Institute of Molecular and Clinical Ophthalmology, Basel, Switzerland; 6 Privatpraxis Prof Jonas und Dr Panda-Jonas, Heidelberg, Germany; 7 Beijing Institute of Ophthalmology, Beijing Tongren Eye Center, Beijing Tongren Hospital, Capital University of Medical Science, 1 Dongjiaomin Lane, Dongcheng District, Beijing, 100730 China

**Keywords:** Eye diseases, Retinal diseases

## Abstract

The study aims to assess the thickness of the retinal outer nuclear layer (ONL), ellipsoid zone (EZ) and photoreceptor outer segment (POS) band in various macular regions and its associations with axial length and other parameters. Participants of the Beijing Eye Study 2011 underwent a series of examinations including spectral-domain optical coherence tomography of the macula. The current study included 2213 participants without retinal or optic nerve diseases (age: 61.7 ± 8.4 years; range 50–93 years); axial length: 23.15 ± 0.95 mm; range 18.96–29.15 mm). The ONL (fovea: 98.9 ± 8.8 µm), EZ (fovea: 24.1 ± 0.5 µm) and POS band (fovea: 24.3 ± 3.5 µm) were the thickest (*P* < 0.001) in the fovea (defined as the thinnest central point), followed by the temporal inner, nasal inner, inferior inner, superior inner, inferior outer, temporal outer, nasal outer, and superior outer region. In multivariable analysis, a thicker retinal ONL was associated (correlation coefficient r: 0.40) with shorter axial length (beta: − 0.14; *P* < 0.001) and shorter disc–fovea distance (beta: − 0.10; *P* = 0.001), after adjusting for younger age (beta: − 0.26; *P* < 0.001), male sex (beta: 0.24; *P* < 0.001), lower serum cholesterol concentration (beta: − 0.05; *P* = 0.04), and thicker subfoveal choroidal thickness (beta: 0.08; *P* < 0.001). The POS thickness increased with shorter axial length (beta: − 0.06; *P* < 0.001) and shorter optic disc–fovea distance (beta: − 0.05; *P* = 0.03), after adjusting for younger age (beta: − 0.34; *P* < 0.001), male sex (beta: 0.15; *P* < 0.001), and thicker subfoveal choroidal thickness (beta: 0.24; *P* < 0.001). As a conclusion, the photoreceptor ONL, EZ and POS band vary in thickness between different macular regions and differ in their correlations with axial length, disc–fovea distance, age, sex, and subfoveal choroidal thickness. The ONL thickness decrease with longer axial length and longer disc–fovea distance may point to an axial elongation-associated retinal stretching in the macula.

## Introduction

Myopic axial elongation is associated with a marked enlargement of the surface of Bruch’s membrane (BM)^1^. Assuming that the retinal photoreceptors cannot undergo mitosis in adolescence or later, myopic axial elongation may lead to a decrease in the photoreceptor density in axially elongated eyes. Previous studies suggested that retinal thickness and density of the retinal pigment epithelium (RPE) cells and choriocapillaris in the macular region and best corrected visual acuity in eyes without myopic maculopathy are independent of axial length^2–6^. In contrast, retinal thickness and RPE cell density decreased with longer axial length in the midperiphery of the fundus^2,3^. We here examined, whether the thickness of the outer nuclear layer (ONL) and the thickness of the ellipsoid zone (EZ) and photoreceptor outer segment (POS) band are correlated with axial length. Although variations in the length of Henle´s nerve fibers can markedly influence the intraregional relationship between the photoreceptor outer segment density and the ONL thickness, the photoreceptor density may be considered to roughly correlate with the ONL thickness^7^. The thickness of the ONL, together with the thickness of the ellipsoid zone (EZ) and photoreceptor outer segment (POS), may thus be considered to be an approximate surrogate for the photoreceptor density in normal eyes^7^. We additionally assessed associations between the retinal outer layer thickness and parameters other than axial length, since the physiological determinants of the thickness of the ONL, EZ and POS have not been fully explored yet.

## Methods

The population-based Beijing Eye Study 2011 was performed in a rural area and an urban region in Greater Beijing^8,9^. The study design was approved by the Ethics Committee of the Beijing Tongren Hospital. All individuals participating in the study gave their written informed consent, and all methods were performed in accordance with the relevant guidelines and regulations. An age of 50+ years and living in the study regions were the only criteria to be fulfilled for being included into the study. Out of 4403 eligible individuals with an age of 50+ years, 3468 subjects (1963 (56.6%) women) were enrolled with a mean age of 64.6 ± 9.8 years (median, 64 years; range 50–93 years).

Since the present study was focused on normal eyes, we excluded for the present investigation eyes with any retinal or optic nerve disorder. Criteria to be fulfilled for inclusion into the present study were a best corrected visual acuity of ≥ 20/25 for eyes with a refractive error ranging between + 1.00 diopter and − 4.00 diopters, and a best corrected visual acuity ≥ 20/33 in eyes with a refractive error of less than − 4.00 diopters. The quality score of the macula OCT scans had to be ≥ 15.

All study participants underwent a detailed interview, biochemical analysis of blood samples, blood pressure measurement, refractometry, photography of the anterior and posterior ocular segment, and optical low-coherence reflectometry (Lenstar 900® Optical Biometer, Haag-Streit, 3098 Koeniz, Switzerland). Using the fundus photographs, we measured the optic disc–fovea distance^10^.

**Figure 1:**
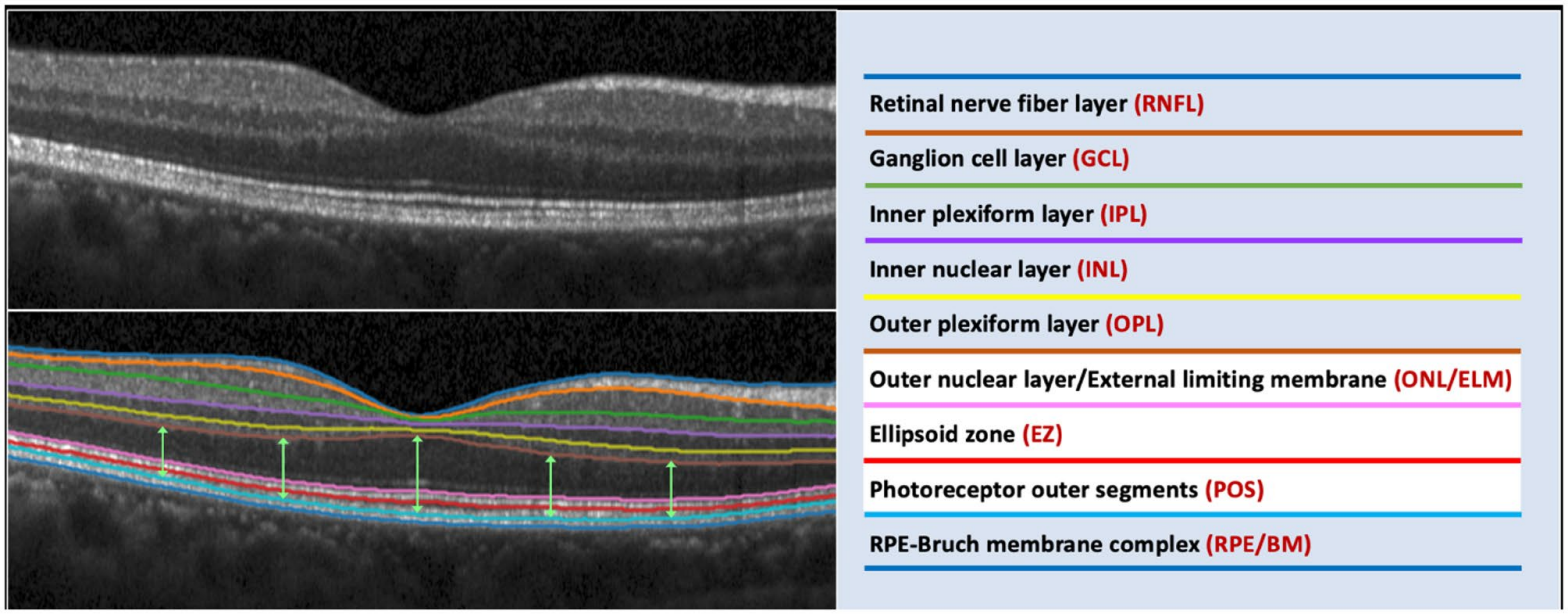
Graph showing the automatic delineation of the nine retinal layers and bands by optical coherence tomography and a deep learning and an interior point method-based algorithm. Nine layers of the retina were segmented, included the retinal nerve fiber layer (RNFL), the ganglion cell layer (GCL), the inner plexiform layer (IPL), the inner nuclear layer (INL), the outer plexiform layer (OPL), the outer nuclear layer/external limiting membrane (ONL/ELM), the ellipsoid zone (EZ), the photoreceptor outer segments band (POS), and the retinal pigment epithelium-Bruch membrane (RPE/BM). The current study focused on the photoreceptor layer, which was composed of three structures, including ONL, EZ and POS, shown as arrow (left figure), and the zones in white background (right figure).

All study participants were examined by spectral-domain OCT (Spectralis OCT; Heidelberg Engineering, Heidelberg, Germany). The macula area was scanned by a cube scan mode, comprising 31 horizontal B scans. The whole scanning area was 30° × 25° centered on the fovea. The depth resolution of the images was 3.87 µm pixel. Each scan line was based on 100 averaged scans. For segmentation of the various retinal layers and bands, we used a multiple-surface OCT segmentation algorithm, which had been developed based on a deep learning approach and an interior point method. The algorithm has been demonstrated to have a test error of less than one-pixel size^11^. After the surfaces of 9 retinal layers and bands had been segmented, the thickness of these 9 layers and bands were computed as the distance between adjacent two surfaces perpendicular to the retinal pigment epithelium (RPE)-BM’s bottom surface (Fig. 1). The 9 layers and bands included the retinal nerve fiber layer (RNFL), the ganglion cell layer (GCL), the inner plexiform layer (IPL), the inner nuclear layer (INL), the outer plexiform layer (OPL), the ONL (including Henle’s fiber layer, outer nuclear layer, external limiting membrane, and myoid zone), the ellipsoid zone (EZ), the photoreceptor outer segment band (POS), and the RPE/BM (Fig. 1)^12,13^. The mean thickness of each layer/band was assessed in 9 macular areas based on the Early Treatment of Diabetic Retinopathy Study map^14^. The foveal center was defined as the thinnest central point. Subfoveal choroidal thickness was measured on the horizontal OCT section running through the center of the fovea and was defined as the vertical distance from the hyperreflective line of the BM to the hyperreflective line of the inner surface of the sclera. The measurements were performed using the Heidelberg Eye Explorer software (v. 5.3.3.0; Heidelberg Engineering Co, Heidelberg, Germany).

For the statistical analysis, we applied a software package (SPSS for Windows, version 27.0; IBM-SPSS, Chicago, IL, USA). We calculated the mean values and the standard deviations of the main outcome parameters, i.e., the thickness of the ONL, EZ and POS. In a second step, we performed univariable analyses to examine associations between the main outcome variables and other ocular and systemic parameters. In a third step, we carried out multivariable analyses which included all independent parameters which were significantly correlated with the main outcome parameters in the univariable analysis. In a step-by-step procedure, we dropped those variables which either exhibited a high collinearity (assessed by the variance inflation factor (VIF)), or that were no longer significantly correlated with the RNFL thickness. We calculated the 95% confidence intervals, the standardized correlation coefficient beta and the non-standardized coefficient B. We considered a two-sided *P*-value of < 0.05 as statistically significant.

## Results

The study included 2213 eyes of 2213 participants (1254 (56.7%) women) with a mean age of 61.7 ± 8.4 years (median: 60 years; range 50–93 years) and mean axial length of 23.15 ± 0.95 mm (median: 23.08 mm; range 18.96–29.15 mm). The participants of the present study as compared with the other individuals taking part in the Beijing Eye Study but not in the present investigation were significantly younger (61.7 ± 8.4 years versus 69.8 ± 9.9 years; *P* < 0.001) and had a significantly longer axial length (23.15 ± 0.95 mm versus 23.47 ± 1.42 mm; *P* < 0.001), while both groups did not differ significantly in sex (men/women: 959/1254 versus 546/709; *P* = 0.94).

**Figure 2:**
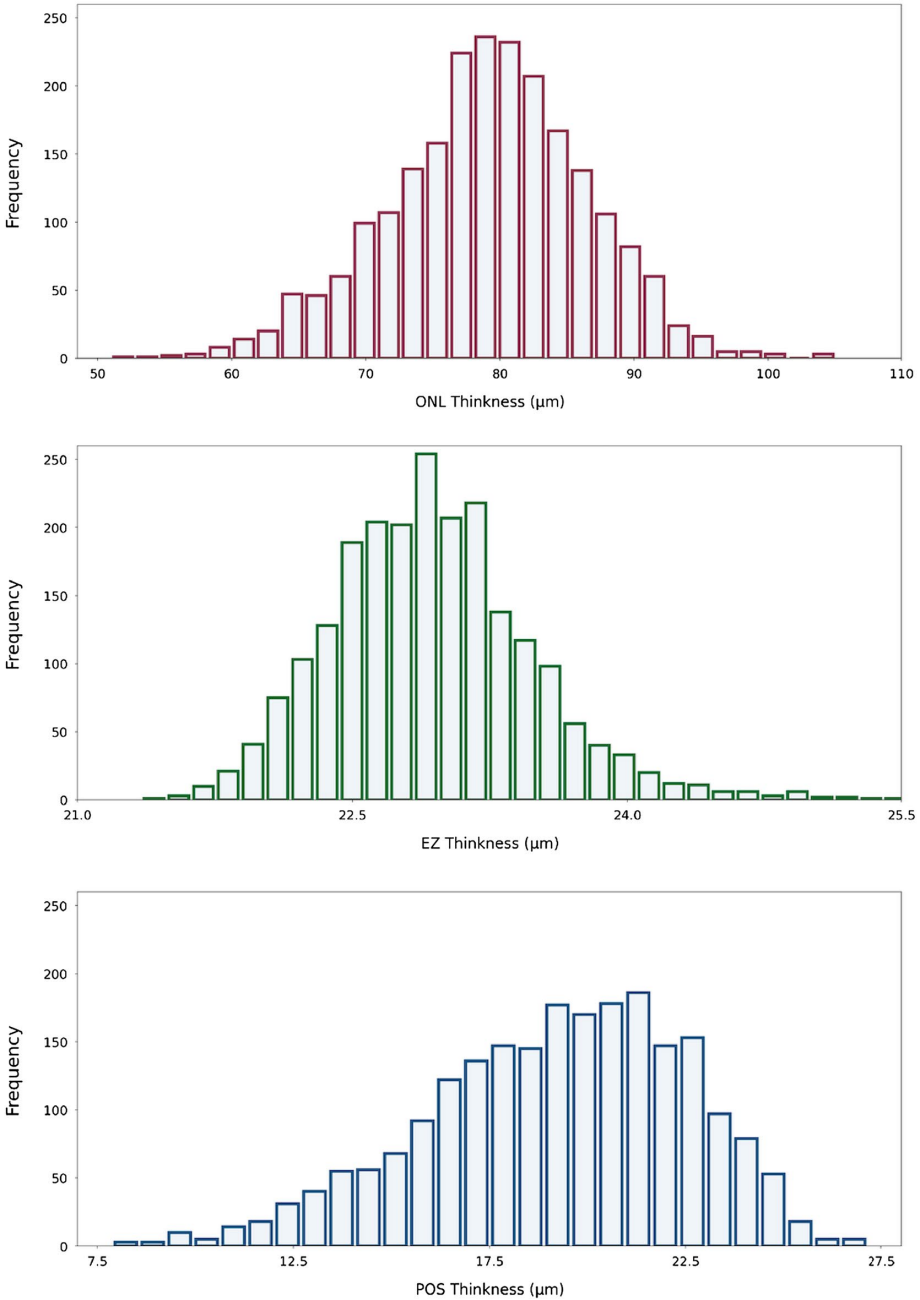
Graph showing the distribution of the thickness of the retinal outer nuclear layer (ONL), the ellipsoid zone (EZ), and the photoreceptor outer segment band (POS) in the Beijing Eye Study.

The mean ONL thickness was 98.9 ± 8.8 µm (median: 99.5 µm; range 69.1–128.2 µm) in the foveal region (Table 1, Fig. 2). The thickness measurements were the thickest (*P* < 0.001) in the fovea, followed by the temporal inner region, in which it was thicker (*P* < 0.001) than in the nasal inner region, the inferior inner region (*P* < 0.001), and the superior inner region. In the latter, it was significantly thicker than in the inferior outer region (*P* < 0.001), followed by the temporal outer region (*P* < 0.001), the nasal outer region (*P* < 0.001), and the superior outer region.

**Table 1:** Thickness of the retinal outer nuclear layer (ONL), the ellipsoid zone (EZ), the photoreceptor outer segment band (POS) and the total of all three structures in 9 Early Treatment Diabetic Retinopathy Study (ETDRS) subfields, in normal participants from the Beijing Eye Study.

	ONL	EZ	POS	Total
Fovea	98.9 ± 8.8	24.1 ± 0.5	24.3 ± 3.5	147.2 ± 10.6
Nasal inner region	84.1 ± 12.6	23.0 ± 0.7	20.9 ± 3.5	128.0 ± 15.1
Nasal outer region	70.7 ± 9.6	22.2 ± 0.8	17.3 ± 3.7	110.1 ± 12.1
Temporal inner region	85.3 ± 7.0	23.1 ± 0.7	20.5 ± 3.3	128.8 ± 8.7
Temporal outer region	73.2 ± 6.2	22.5 ± 1.0	17.3 ± 3.6	113.1 ± 8.1
Superior inner region	77.9 ± 9.1	22.9 ± 0.7	19.1 ± 3.6	119.9 ± 11.5
Superior outer region	65.1 ± 7.0	23.4 ± 1.3	14.9 ± 4.1	103.3 ± 9.3
Inferior inner region	83.0 ± 8.1	22.3 ± 0.7	20.5 ± 3.5	126.4 ± 10.4
Inferior outer region	73.5 ± 6.9	22.5 ± 1.0	18.9 ± 3.9	114.1 ± 9.0
Mean	79.1 ± 7.4	22.9 ± 0.6	19.2 ± 3.3	121.2 ± 9.5

Data are presented as mean ± standard deviation (µm).

In a similar manner, the EZ (foveal region: 24.1 ± 0.5 µm) was the thickest in the fovea (*P* < 0.001), followed by the superior outer region (*P* < 0.001), temporal inner region (*P* < 0.001), nasal inner region (*P* < 0.001), superior inner region (*P* < 0.001), and the inferior outer region (*P* < 0.001). The latter region did not differ significantly from the temporal outer region, where it was thicker than inferior inner region (*P* < 0.001), and finally the nasal outer region (*P* < 0.001).

The POS thickness (foveal region: 24.3 ± 3.5 µm) was the largest in the fovea (*P* < 0.001), followed by the nasal inner region, the inferior inner region (*P* < 0.001), the temporal inner region (*P* = 0.005), the superior inner region (*P* < 0.001), the inferior outer region (*P* < 0.001), the temporal outer region (*P* < 0.001) and the nasal outer region(*P* < 0.001) (without a significant difference between both regions (*P* = 0.87),), and finally the superior outer region.

The total thickness of all three structures together (ONL, EZ, POS) also was the thickest in the foveal region (147.2 ± 10.6 µm) (*P* < 0.001), followed by the temporal inner region, nasal inner region (*P* = 0.001), the inferior inner region (*P* < 0.001), the superior inner region (*P* < 0.001), the inferior outer region (*P* < 0.001), the temporal outer region (*P* < 0.001), the nasal outer region (*P* < 0.001), and finally the superior outer region (*P* < 0.001).

**Figure 3:**
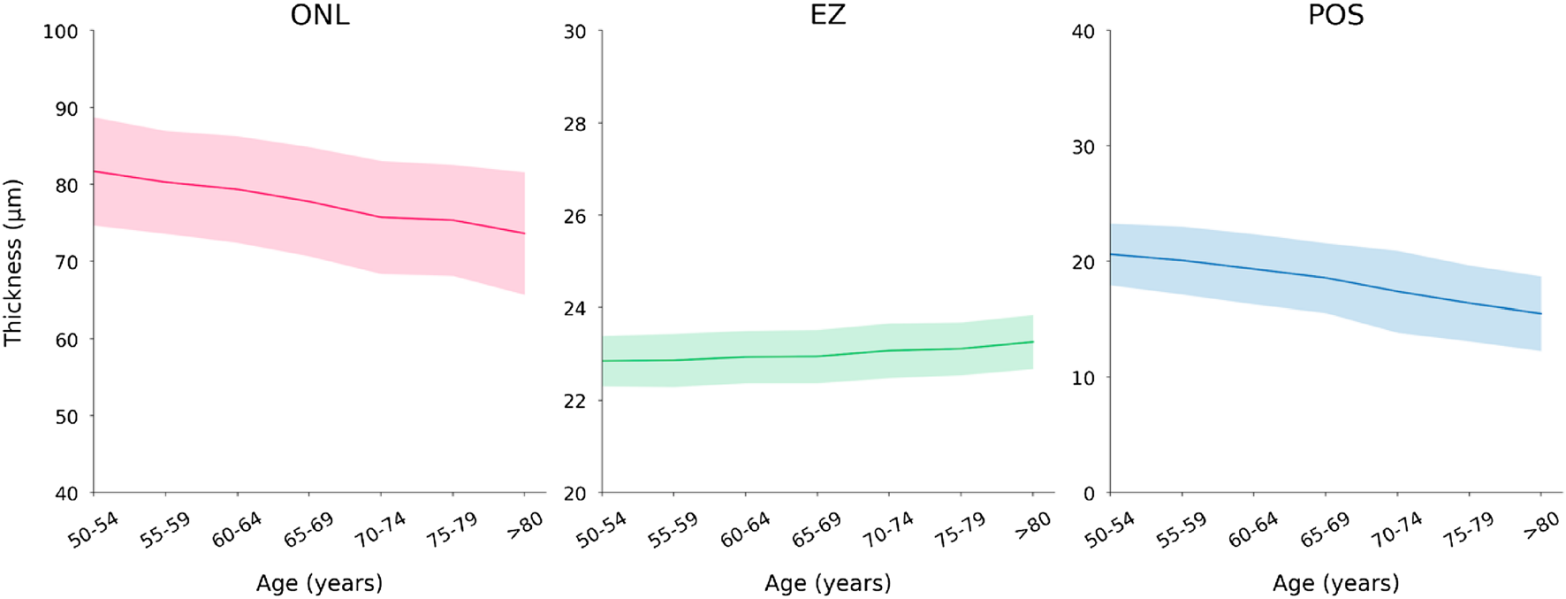
Graph showing the age-related change (mean value (line) and standard deviation (shadow)) in the thickness of retinal outer nuclear layer (ONL), ellipsoid zone (EZ), and photoreceptor outer segment band (POS).

**Figure 4:**
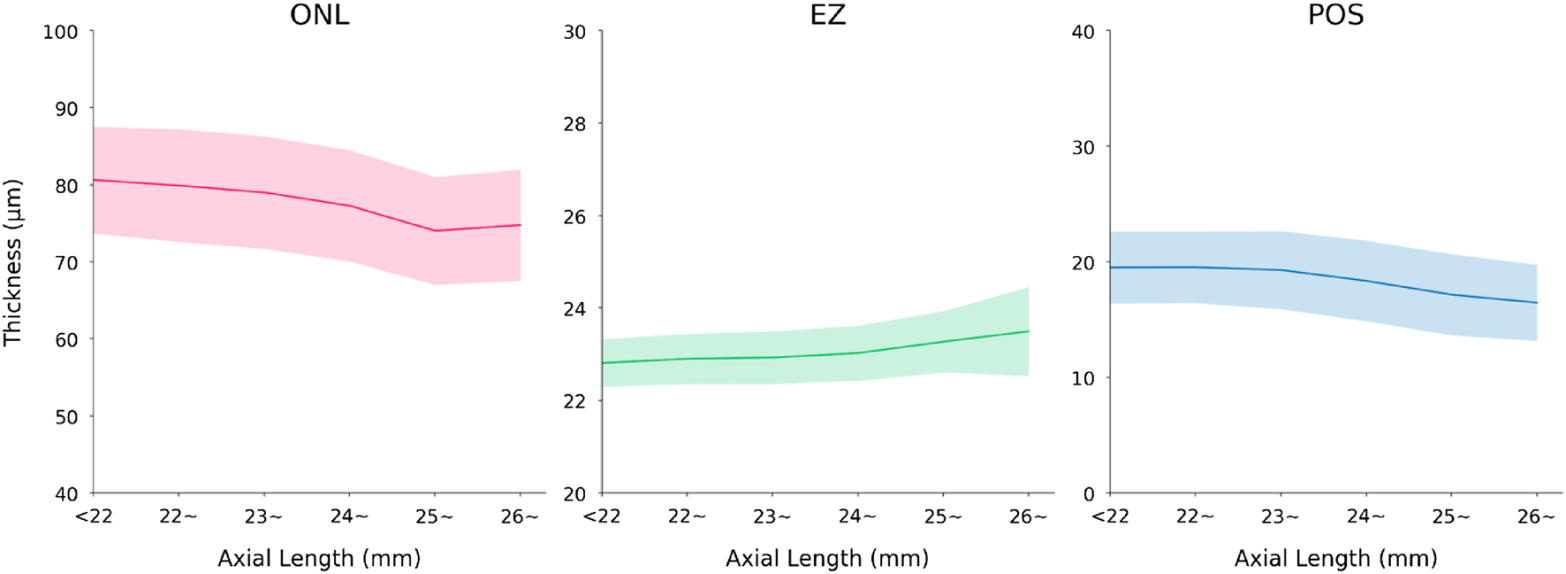
Graph showing the axial length-related change (mean value (line) and standard deviation (shadow)) in the thickness of the retinal outer nuclear layer (ONL), ellipsoid zone (EZ), and photoreceptor outer segment band (POS).

In univariable analysis, a thicker ONL thickness (as mean of all macular regions) correlated with parameters such as younger age (Fig. 3) and shorter axial length (Table 2, Fig. 4). In the multivariable analysis, a thicker retinal ONL as mean of all macular regions, was associated (correlation coefficient r: 0.40) with younger age (*P* < 0.001), male sex (*P* < 0.001), lower serum concentration of cholesterol (*P* = 0.04), shorter axial length (*P* < 0.001), shorter disc–fovea distance (*P* = 0.001), and thicker subfoveal choroidal thickness (*P* < 0.001) (Figs. 3, 4, Table 3).

**Table 2:** Associations between the thickness of the retinal outer nuclear layer (ONL), the ellipsoid zone (EZ), the photoreceptor outer segment band (POS), with ocular and systemic parameters in a univariable analysis.

	ONL		EZ		POS	
	P	Correlation coefficient	P	Correlation coefficient	P	Correlation coefficient
Age (years)	< 0.001	− 0.31	< 0.001	0.17	< 0.001	− 0.43
Gender (men/women)	< 0.001	− 0.16	< 0.001	0.10	< 0.001	− 0.10
Rural/urban region	< 0.001	− 0.21	0.005	0.06	< 0.001	− 0.13
Level of education	< 0.001	− 0.08	0.71	0.01	0.29	− 0.02
Body height (cm)	< 0.001	0.13	< 0.001	− 0.10	< 0.001	0.14
Body weight (kg)	< 0.001	0.10	0.001	− 0.07	< 0.001	0.10
Body mass index (kg/m^2^)	0.186	0.03	0.36	− 0.02	0.26	0.02
Waist circumference (cm)	0.162	0.03	0.47	− 0.02	0.89	0.00
Cognitive score	0.006	0.06	0.20	− 0.03	0.001	0.07
Depression score	0.50	0.01	0.98	0.00	0.77	− 0.01
Alcohol consumption quantity	< 0.001	0.15	< 0.001	− 0.11	< 0.001	0.14
Smoking package years	< 0.001	0.13	0.001	− 0.07	0.001	0.07
Blood pressure, systolic (mmHg)	0.11	− 0.03	0.09	0.04	< 0.001	− 0.10
Blood pressure, diastolic (mmHg)	< 0.001	0.08	0.19	− 0.03	0.005	0.06
Blood concentrations of						
Glucose (mmol/L)	0.03	− 0.05	0.003	− 0.07	0.015	− 0.06
Cholesterol (mmol/L)	0.02	− 0.06	0.31	− 0.03	0.26	0.03
High-density lipoproteins (mmol/L)	0.92	0.00	0.76	0.01	0.09	0.04
Low-density lipoproteins (mmol/L)	0.21	− 0.03	0.45	− 0.02	0.13	0.04
Triglyceride (mmol/L)	0.04	− 0.05	0.19	− 0.03	0.27	− 0.03
Creatinine (mmol/L)	0.68	0.01	0.49	0.02	0.03	− 0.07
Diabetes mellitus, prevalence	0.054	− 0.04	0.03	− 0.05	< 0.001	− 0.08
Arterial hypertension, prevalence	0.01	− 0.06	0.61	0.01	< 0.001	− 0.09
Ocular parameters						
Spherical equivalent (Diopters)	0.04	0.05	< 0.001	− 0.18	< 0.001	0.08
Intraocular pressure (mm Hg)	< 0.001	0.07	0.11	− 0.03	< 0.001	0.08
Central corneal thickness (µm)	0.90	0.00	0.07	− 0.04	0.217	0.03
Corneal curvature radius (mm)	< 0.001	− 0.13	0.11	0.04	0.02	− 0.05
Axial length (mm)	< 0.001	− 0.18	< 0.001	0.16	< 0.001	− 0.15
Optic disc–fovea distance (mm)	< 0.001	− 0.15	< 0.001	0.12	< 0.001	− 0.10
Choroidal thickness (µm)	< 0.001	0.27	< 0.001	− 0.29	< 0.001	0.40

The thickness of each layer/band was calculated as the mean value of all 9 Early Treatment Diabetic Retinopathy Study (ETDRS) subfields.

**Table 3:** Associations between the thickness of the retinal outer nuclear layer (ONL) with ocular and systemic parameters in a multivariable analysis.

	Standardized coefficient (beta)	Non-standardized coefficient (B)	95% CI of B	*P*-value	VIF
Age (years)	− 0.26	− 0.24	− 0.28, − 0.19	< 0.001	1.15
Sex (female/male)	0.24	3.48	2.75, 4.22	< 0.001	1.20
Serum cholesterol (mmol/L)	− 0.05	− 0.37	− 0.73, − 0.02	0.04	1.05
Axial length mm)	− 0.14	− 1.15	− 1.63, − 0.67	< 0.001	1.63
Disc–fovea distance (mm)	− 0.10	− 2.28	− 3.59, − 0.97	0.001	1.46
Choroidal thickness (µm)	0.08	0.006	0.002, 0.010	0.004	1.24

The layer thickness was calculated as the mean value of all 9 Early Treatment Diabetic Retinopathy Study (ETDRS) subfields.

*CI* confidence interval, *VIF* variance inflation factor.

As the overall ONL thickness, so were the regional ONL thickness measurements associated mostly with the same parameters. The foveal ONL thickness increased with younger age (beta: − 0.21; *P* < 0.001), male sex (beta: 0.24; *P* < 0.001), lower serum concentration of cholesterol (beta: − 0.05; *P* = 0.04), shorter axial length (beta: − 0.06; *P* < 0.001), shorter disc–fovea distance (beta: − 0.17; *P* = 0.001), and thicker subfoveal choroidal thickness (beta: 0.07; *P* < 0.001). If the parameter of sex was replaced by the parameter of body height, the latter was significantly associated with a thicker ONL (beta: 0.19; B: 0.18; 95% CI 0.13, 0.23; *P* < 0.001). In particular with respect to axial length and disc–fovea distance, all regional ONL thickness measurements correlated with shorter axial length and shorter disc–fovea distance.

In contrast to the overall ONL, a thicker EZ as mean of all macular regions was associated (correlation coefficient r: 0.32) with older age (beta: 0.09; *P* < 0.001), female sex (beta: 0.15; *P* < 0.001), longer axial length (beta: 0.08; *P* = 0.005), longer optic disc–fovea distance (beta: 0.05; *P* = 0.04), and thinner subfoveal choroid (beta: − 0.21; *P* < 0.001) (Figs. 3, 4, Table 4). When the parameter of subfoveal choroidal thickness was dropped, the results of the other associations remained mostly unchanged.

**Table 4:** Associations between the thickness of the ellipsoid zone (EZ) with ocular and systemic parameters in a multivariable analysis.

	Standardized coefficient (beta)	Non-standardized coefficient (B)	95% CI of B	*P*-value	VIF
Age (years)	0.09	0.006	0.003, 0.009	< 0.001	1.17
Sex (male/female)	0.13	0.15	0.10, 0.20	< 0.001	1.13
Axial length mm)	0.08	0.05	0.02, 0.08	0.005	1.70
Disc–fovea distance (mm)	0.05	0.10	0.004, 0.19	0.04	1.52
Choroidal thickness (µm)	− 0.21	− 0.001	− 0.001, − 0.001	< 0.001	1.29

The thickness was calculated as the mean value of all 9 Early Treatment Diabetic Retinopathy Study (ETDRS) subfields.

*CI* confidence interval, *VIF* variance inflation factor.

As the overall EZ thickness, so were the regional EZ thickness measurements associated with similar parameters. The foveal EZ thickness increased with younger age (beta: − 0.18; *P* < 0.001) and thinner subfoveal choroidal thickness (beta: − 0.12; *P* < 0.001), while it was not significantly related with axial length (*P* = 0.48), disc–fovea distance (*P* = 0.07) and sex (*P* = 0.24).

As it was the case with the POS thickness, its average thickness increased with younger age (beta: − 0.34; *P* < 0.001), male sex (beta: 0.15; *P* < 0.001), shorter axial length (beta: − 0.06; *P* < 0.001), shorter optic disc–fovea distance (beta: − 0.05; *P* = 0.03), and thicker subfoveal choroidal thickness (beta: 0.24; *P* < 0.001) (Figs. 3, 4, Table 5).

**Table 5:** Associations between the thickness of the photoreceptor outer segment band (POS), with ocular and systemic parameters in a multivariable analysis.

	Standardized coefficient (beta)	Non-standardized coefficient (B)	95% CI of B	*P*-value	VIF
Age (years)	− 0.34	− 0.14	− 0.15, − 0.12	< 0.001	1.17
Sex (female/male)	0.15	0.99	1.26, 0.73	< 0.001	1.13
Axial length mm)	− 0.06	− 0.20	− 0.37, − 0.02	< 0.001	1.70
Disc–fovea distance (mm)	− 0.05	− 0.55	− 1.04, − 0.07	0.03	1.53
Choroidal thickness (µm)	0.24	0.08	0.006, 0.009	< 0.001	1.29

The band thickness was calculated as the mean value of all 9 Early Treatment Diabetic Retinopathy Study (ETDRS) subfields.

*CI* confidence interval, *VIF* variance inflation factor.

## Discussion

In our population-based study on normal eyes, ONL thickness in the fovea and the perifoveal macular regions was associated with shorter axial length and shorter disc–fovea distance in a multivariable analysis. These observations agree with the findings of other investigations. In the study population of the UK biobank, the combined thickness of the layers from the INL to the RPE increased with younger age, male sex, non-Black ethnicity, no smoking, lower systolic blood pressure, more hyperopic refractive error, higher intraocular pressure and higher corneal hysteresis^15^. In this comparison it should be taken into account that the UK Biobank included different anatomic metrics from what was measured in our study (combined thickness of the layers from the INL to the RPE versus ONL thickness). With respect to the disc–fovea distance, a parameter not examined in the UK biobank study, Qiu and colleagues reported that it was reversely correlated with the mean thickness of all intraretinal layers of the macula except for the retinal ganglion cell layer and the layer with photoreceptors^16^. It is partially in contrast to the results of our study in which the ONL thickness was negatively, the EZ thickness was positively, and the POS thickness was negatively correlated with a longer disc–fovea distance (Tables 3, 4, 5).

The association between a decreasing ONL thickness and older age is in agreement with histomorphometric studies in which the count of retinal photoreceptors (as well as the count of the retinal pigment epithelium cells) and the number of optic nerve fibers decreased by about 0.3% per year of age^17–20^. The association between a thicker ONL and male sex after adjusting for other variables such as age and axial length in the current investigation as well as in the UK biobank study corresponded to the relationship between taller body height and a thicker ONL. Correspondingly, if the parameter of sex was substituted by the parameter of body height, taller body correlated with a thicker ONL (beta: 0.19; *P* < 0.001).

Of special interest is the relationship between the thickness of the retinal layers and axial length and disc–fovea distance. In the previous UK biobank study as in the present investigation longer eyes (beta: − 0.14) and, independently of axial length, eyes with a longer disc–fovea distance (beta: − 0.10) had a thinner average ONL. The same observations were made for the foveal ONL, with a higher amount of the regression coefficient for the disc fovea distance (beta: − 0.16) than for axial length (beta: − 0.08). Previous histological studies have suggested that BM in the macular region may not undergo marked changes during axial elongation in eyes with myopic maculopathy (stage 3 or higher)^1^. Reasons for this assumption were that BM thickness did no decrease with longer axial length, and that the length of BM, measured from the foveal center to the temporal border of parapapillary gamma zone was independent of axial length^21,22^. The axial elongation-associated increase in the disc–fovea distance was due to the development and enlargement of parapapillary gamma zone^22,23^. Correspondingly, the RPE density and the choriocapillaris, as measured histomorphometrically, were not associated with axial length^3,4^. The hypothesis that axial elongation is mainly due to an enlargement of BM in the midperiphery of the fundus explains the axial elongation-associated thinning of the retina and decrease in the RPE density in that location. BM in the macular region however does not increase in size with longer axial length. It is in contrast to the finding that the ONL thickness in the macular region decreased with longer axial length. A potential reason for this discrepancy could be the axial elongation-related enlargement of the retinal surface. It might result in an increased strain within the tissue, resulting in a thinning of the retina, most marked in the fundus midperiphery, and to a minor amount, in the macular region. This decrease in the macular ONL thickness was not pronounced as indicated by the relatively low standardized regression coefficient of beta =  − 0.14 (Table 3). In that model, the correlation between a longer disc–fovea distance and a thinner ONL, independently of the axial length, may be due to an additional retinal stretching effect in the macular region, with the disc–fovea distance as a surrogate for this regional enlargement of the inner ocular surface in the macular region. It has remained unclear, why the EZ thickness increased with longer axial length and longer disc–fovea distance, in contrast to the ONL thickness and the thickness of the photoreceptor outer segments (Tables 3, 4, 5).

Our study´s findings on a decrease in the macular ONL thickness with longer axial length concur with results of studies applying adaptive optics scanning laser ophthalmoscopy. These investigations reported that the density of the foveal cones was lower in eyes with longer eyes. The cone density was, however, not linearly correlated with axial length^24–28^. From a point of view of physical optics, the axial length-associated decrease in the foveal cone density (assessed as cells/mm^2^) was more than compensated by the axial elongation-related increase in the size of the optical image, so that the foveal cone angular sampling density (assessed as cells/deg^2^) was higher in eyes with longer axial length. Eckmann-Hansen et al. reported that the absolute cone density was reduced by 9.7% for an axial elongation by 1 mm. In contrast, the angular cone density decreased by only 0.84% for an axial elongation by 1 mm^28^. These findings are corroborated by reports that the best corrected visual acuity was independent of axial length in eyes with myopic maculopathy^6^, and they support the hypothesis.

The reasons for the relationship between a thicker ONL and male sex, or between a thicker ONL and taller body height have remained unclear. In previous studies, males versus females tended to have larger globes including a longer axial length (without necessarily being myopic), and also taller body height was correlated with a larger globe size (longer axial length)^29,30^. To cite an example, in the population-based Ural Eye and Medical Study, longer axial length correlated with taller body height after adjusting for parameters such as age, level of education, myopic spherical refractive error, corneal refractive power, anterior chamber depth^29^. In the Singapore Indian Eye Study, men versus women had significantly longer axial length, and longer axial length was associated with taller body height after adjusting for educational level, and time spent with reading^30^. Correspondingly, the number of retinal photoreceptors and retinal ganglion cells (as measured by the optic nerve fibers) increased with larger retinal surface area, i.e., with a larger globe size, in a histomorphometric study^31^. The observations of the physiological associations of the thickness of the ONL, EZ and POS with other parameters such as age, axial length and disc–fovea distance may be useful for the assessment of associations between the thickness of these structures and diseases, such as age-related macular degeneration, as shown in a recent study by Trinh et al. and by Zekavat et al.^32,33^.

When the findings of our study are discussed, its limitations have to be taken into account. First, due to the low visibility of external limiting membrane (ELM), it had not been segmented separately but included into the measurement of the ONL thickness as part of the ONL. Second, the determination of the measurement points on the OCT scans did not account for the steep gradients in photoreceptor cell densities within the macula, so that inaccuracies in determining the measurement point on the single OCT scan may have led to inaccuracies in the layer and band measurements. Third, the “ONL” as measured in our study included not only the nuclei of the photoreceptors but additionally Henle´s fiber layer, the external limiting membrane and the myoid zone, so that, just due to anatomical reasons, it cannot be a pure surrogate for the number of photoreceptors. Any association between the “ONL” and other parameters such as the axial length might have been markedly influenced by structures other than the photoreceptor nuclei, namely Henle´s fibers and the myoid zone. In addition, one may consider that the layer containing the photoreceptor cell nuclei additionally includes interleaved Müller glial processes. Fourth, the beta coefficients of some associations are relatively small, and might have reached statistical significance only because of the relatively large sample size. Fifth, we did not correct for the magnification of the OCT-based fundus image by the optic media of the eye^34^. In the OCT device used in our study (Spectralis®), the image magnification by the ocular media may at least partially be compensated for by taking into account of the keratometric readings of the individual eye. In a previous study conducted by Niyazmand et al., the raw retinal thickness measurements by the Spectralis® were underestimated for the foveal region and overestimating in extrafoveal regions^35^. For each axial length increase by one millimeter, the foveal thickness was overestimated by 2.7 μm to 2.9 μm and the retinal thickness in extrafoveal regions was underestimated by 0.2 μm to 4.1 μm. This potential weakness in our study design may however only serve to strengthen the observation, that increasing axial length correlates with a slight decrease in the photoreceptor density in the fovea.

In conclusion, the photoreceptor ONL, EZ and outer segment layer vary in thickness between different macular regions and differ in their correlations with age, sex, axial length, disc–fovea distance and subfoveal choroidal thickness. The ONL decreases in thickness with longer axial length and longer disc–fovea distance. It may point to a slight axial elongation-associated stretching effect of the retina in the macular region.

## Data Availability

All identified data are available upon reasonable request from the corresponding author.
